# Microvascular Anastomosis Training in Neurosurgery: A Review

**DOI:** 10.1155/2018/6130286

**Published:** 2018-03-28

**Authors:** Vadim A. Byvaltsev, Serik K. Akshulakov, Roman A. Polkin, Sergey V. Ochkal, Ivan A. Stepanov, Yerbol T. Makhambetov, Talgat T. Kerimbayev, Michael Staren, Evgenii Belykh, Mark C. Preul

**Affiliations:** ^1^Irkutsk State Medical University, Krasnogo Vosstaniya St. 1, Irkutsk, Russia; ^2^Irkutsk Scientific Center of Surgery and Traumatology, Bortsov Revolutsii St. 1, Irkutsk, Russia; ^3^Railway Clinical Hospital, Irkutsk-Passazhirskiy of Russian Railways Ltd., Botkina St. 10, Irkutsk, Russia; ^4^Irkutsk State Medical Academy of Postgraduate Education, Jubileiniyi 100, Irkutsk, Russia; ^5^National Center of Neurosurgery, 34/1 Turan Av., Astana, Kazakhstan; ^6^Department of Neurosurgery Research, Barrow Neurological Institute, St. Joseph's Hospital and Medical Center, 350 West Thomas Road, Phoenix, AZ 85013, USA

## Abstract

Cerebrovascular diseases are among the most widespread diseases in the world, which largely determine the structure of morbidity and mortality rates. Microvascular anastomosis techniques are important for revascularization surgeries on brachiocephalic and carotid arteries and complex cerebral aneurysms and even during resection of brain tumors that obstruct major cerebral arteries. Training in microvascular surgery became even more difficult with less case exposure and growth of the use of endovascular techniques. In this text we will briefly discuss the history of microvascular surgery, review current literature on simulation models with the emphasis on their merits and shortcomings, and describe the views and opinions on the future of the microvascular training in neurosurgery. In “dry” microsurgical training, various models created from artificial materials that simulate biological tissues are used. The next stage in training more experienced surgeons is to work with nonliving tissue models. Microvascular training using live models is considered to be the most relevant due to presence of the blood flow. Training on laboratory animals has high indicators of face and constructive validity. One of the future directions in the development of microsurgical techniques is the use of robotic systems. Robotic systems may play a role in teaching future generations of microsurgeons. Modern technologies allow access to highly accurate learning environments that are extremely similar to real environment. Additionally, assessment of microsurgical skills should become a fundamental part of the current evaluation of competence within a microneurosurgical training program. Such an assessment tool could be utilized to ensure a constant level of surgical competence within the recertification process. It is important that this evaluation be based on validated models.

## 1. Introduction

Cerebrovascular diseases are among the most widespread diseases in the world, which largely determine the structure of morbidity and mortality rates. Cerebral vascular pathology comprises the major part of neurology and neurosurgery. Today, significant progress has been made in the field of preventative revascularization of strokes due to the introduction of new technologies of microneurosurgery into clinical practice, contributing to the improvement of safety of treatment. It was shown that cerebral revascularization using extracranial to intracranial bypass may result in neurological symptoms improvement and an objective increase in regional cerebral blood flow in a selective cohort of patients with symptomatic chronic cerebrovascular ischemia [1]. But at the same time, several randomized controlled trials have shown no benefit of surgical revascularization compared to the best medical treatments to prevent stroke [2]. Bypass techniques are still valuable for the surgical treatment of complex cerebral aneurysms by reducing the risk of temporary and constant brain ischemia due to the alteration in blood flow.

Microvascular anastomosis techniques are important for revascularization surgeries on brachiocephalic and carotid arteries, complex cerebral aneurysms, and even during resection of brain tumors that obstruct major cerebral arteries. Successful performance of such complex procedures necessitates advanced requirements for neurosurgical training programs. Such training should include anastomosis practice in the depth of operative wound in conditions of limited vascular mobility and limited time periods.

Because of its complexity, the microanastomosis technique cannot be acquired by observing more experienced surgeons. Training in microsurgery requires considerable time and training resources. Surgeons develop microsurgical skills during training courses or in local hospital laboratories by practicing on various simulation models, using both synthetic and biological materials [3]. Analysis of the training of neurosurgery residents revealed a growing dissatisfaction with the quality of training as forty percent of young surgeons rated their microsurgical training as inadequate [4]. In this regard, neurosurgical training programs may benefit from an increased focus on hands-on microsurgical training.

In this text we will briefly discuss the history of microvascular surgery, review current literature on the simulation models with the emphasis on their merits and shortcomings, and describe the views and opinions on the future of the microvascular training in neurosurgery.

## 2. History of Microanastomosis Techniques Development

Modern surgery cannot be imagined without the vascular suture. The history of surgical interventions on the vessels goes back to at least ancient Hindu civilization, Greece, and Rome [24]. Instructions and experience to perform ligature procedures to locally arrest hemorrhage can be traced from Celsus through Galen, Oribasius, Rhases, Avicenna, Averroes, Guy of Cauliac, Lanfrancus, and Ambrose Pare. Ancient and medieval skilled surgeons or trained medical personnel traveling with the army frequently placed ligatures around bleeding vessels. They knew how to use tourniquets, arterial clamps, and ligatures to stem blood flow. It was common knowledge among ancient surgeons that veins and arteries carried blood; it was obvious to those dealing with horrendous injuries of weapons such as swords, knives, and spears which caused catastrophic peripheral injuries to soldiers and gladiators. The Roman surgeon was highly motivated to keep as many soldiers as functional as possible and had many delicate instruments for dealing with bleeding vessels. Hooks, probes, and other long thin metal instruments allowed efficient and effective maneuvering of small pieces of tissue. Such instruments were primarily used for dissection and for identifying and isolating blood vessels.

The first documented case of vessel recovery was an operation conducted by Hallowell in 1759, in which the patient was sutured with a lateral lesion of the wall of the brachial artery [25]. In 1877 Russian surgeon Eck first performed the anastomosis “side-by-side” between two vessels: the portal and the inferior vena cava [26]. In 1889 Jassinowski used thin nodular sutures when sewing a damaged vessel in a person, suggesting that vascular sutures should not penetrate through the intima of the vessel [27]. However, the most significant technical breakthrough occurred in 1902, when Carrel reported [28] the triangulation technique of anastomosing the vessel for which in part he was awarded the Nobel Prize in 1912. It is this technique of anastomosis developed by Alexis Carrel that is still used today. The following year, Hopfner reported a successful limb replantation in a dog [29]. The work of these pioneers laid the foundation for the subsequent development of microvascular surgery.

Introduction to clinical practice of anticoagulants was one of the key events in vascular surgery. Heparin was discovered by McLean in 1916 and the first successful clinical trials were published in the 1930s [30, 31]. The ability to control clotting was an important step in the development of microvascular surgery.

The final innovation that prompted the beginning of microvascular surgery was the introduction of an operating microscope into clinical practice. In 1954, in Sweden, Nylean experimented on a rabbit fenestration of the ear labyrinth fistula under a microscope with an increase of 10–15 magnification [32]. In 1922, his supervisor Holmgren invented the world's first binocular microscope and introduced its use in otology [33].

In 1963, American neurosurgeon Kurze introduced a microscope into neurosurgical practice to remove the neuroma of the seventh nerve [33, 34]. Then, in 1960 Jacobson and Suarez first described the technique of performing vascular microanastomoses using an operating microscope, which marked the beginning of microvascular surgery [35].

In 1962 Jacobson together with Donaghy reported the first endarterectomy from the middle cerebral artery, using microsurgical reconstruction [36]. In 1965 Potts performed an anastomosis between the superficial temporal artery and the anterior cerebral artery, using a synthetic prosthesis as a shunt [37]. The era of widespread development of revascularization surgery began on October 30, 1967. After a year of training at the Donaghy Microsurgical Laboratory, Yasargil first performed the superposition of an extra-intracranial (EC-IC) microanastomosis between the superficial temporal artery and the cortical branch of middle cerebral artery in a patient with occlusion of the internal carotid artery [38].

Since the 1970s, reconstructive vascular surgeries have begun in the vertebrobasilar basin. Sundt performed EC-IC bypass between occipital artery and posterior cerebral artery in patients with cerebral circulation disorder [39]. Further development of vascular microneurosurgery led to the development of intra-intracranial (IC-IC) microanastomosis techniques. In 1979 Miller et al. conducted a microanastomosis between the middle meningeal artery and the cortical branch of the middle cerebral artery in a 61-year-old patient with parasagittal meningioma [40]. This technique has been intensively developed since the early 1990s and has found its application in reconstructive interventions in the treatment of complex cerebral aneurysms.

## 3. Overview of Definitions

The term validity is borrowed from literature on the problems of knowledge assessment and attestation. Its use in the context of simulation differs from the traditional one. In the context of knowledge assessment, in the simplest form, validity reflects the adequacy of the methodology in measuring outcomes. With respect to simulation, validity is the ability to teach higher cognitive, emotional, and psychomotor skills as much as is expected by the degree of realism achieved.

To assess the simulation models, several types of validity are distinguished [3]. “Face validity” is used to determine the realism of the situation and shows how well the experimental model reflects the actual operation; “content validity” is an assessment of the adequacy of the simulation, to what extent the model estimates a specific skill, and not other aspects, for example, anatomical knowledge; “construct validity” determines whether the successful passage of the model correlates with real operational skills, thus differentiating beginners and experts; “predictive validity” determines that the skills acquired in the simulator training process reflect the skill level in the real operating theater and are translated into clinical practice [41].

## 4. Simulation Models of Microanastomosis for a “Dry” Training

Among the various models used for the training of the microanastomosis skills (Table 1), dry models are the simplest ones.

In “dry” microsurgical training, simulation models simulating biological tissues created from various materials are used. Such models are useful for beginning surgeons, since they are economically viable and do not require sophisticated technical equipment or coordination with an ethical committee. Disadvantages include low face and content validity. Thus, such models are only suitable for practicing basic skills of microscopy, training beginning surgeons with the subsequent transition to living models, and maintaining the skill of more experienced surgeons [42].

At the initial stage of the microvascular training, silicone tubes are used, which allow for the explanation of the basic principles of superposition of different types of anastomoses [43]. They are available in the form of cards with a set of tubes of different diameters: 2.0 mm, 1.0 mm, and 0.7 mm. In addition to silicone, the tubes can also be made of polyurethane [5], a thin latex [44], or hydrogel based on polyvinyl alcohol [45]. A simple model of microanastomosis can also be made from a conventional surgical glove [6]. This model is created by two longitudinal incisions of the glove strip, in which the glove site is then sewn by nodal seams. The obtained tube of glove rubber is suitable for testing anastomosing technique.

Such models can have potentially high construct validity. However, they have low face validity in view of the lack of similarity with living tissues since the elastic properties of the tubes do not correspond to the properties of living vessels. Thus, they cannot fully replace the “wet” training. With silicone tubes, it is best to train counter-pressure technique, suturing, and knotting in different directions.

To develop skills of anastomosing in a deep field, advanced synthetic models with an artificial skull and brain can be used, allowing for the use of brain retractors [46]. Modeling of the topographic location of arteries and cranial structures is carried out with computed tomography data and may be produced for a specific patient. Such models are excellent for sharpening the skills of working with microinstruments in the deep surgical field, handholding techniques with the hand rest against the cranial structures, and exercising suturing on vessels located with variable depths [47].

## 5. Tissue Simulation Models for a “Wet” Training

The next stage in training more experienced surgeons is to work with nonliving biological tissue models. Similarly to artificial models, nonliving biological models do not require complex technical equipment, coordination with the ethics committee (except for human tissues), and the availability of laboratories and have a relatively low cost. They have a potentially high face and construct validities, as they provide experience of handling of biological tissue. The main drawback of such models is the absence of living tissues and functioning blood flow.

The simplest tissue model is the use of chicken blood vessels taken from the wing [48]. Some authors reported that the use of chicken wing arteries reduced the use of live models by 80–100% [49]. The average size of the cortical branch of the middle cerebral artery is 1 mm (range 0.6–1.4 mm), which corresponds to the size of the radial arteries of the chicken wings [50]. A more efficient alternative to a chicken vessels can be the use of turkey wings [7]. Comparative studies show the superiority of turkey vessels due to the larger size of the vessels and decreased variability [51]. Other vessels exist such as in the thigh, but these require dissection, and such sections of the chicken or turkey are more expensive as they are more preferred food grade product. Chicken and turkey wings are seldom preferred for consumption and thus are much less cost to obtain. The vessels at the top of the wing are easily identified and dissected, and many wings can be stored in the freezer and then thawed and used by the student when convenient for practice.

The technique of microvascular anastomosis can also be reproduced using human or bovine placentas [9]. Human placenta anastomosis model includes cleaning of the fetal surface of the placenta, isolation of a second, or third-order arterial branch of 2-3 mm in diameter and more than 30 mm in length. This branch is then dissected free, cut distally, and moved towards the adjacent 1-2 mm arterial branch to perform an end-to-side anastomosis. Human placental arteries and bovine placental veins are convenient, are anatomically relevant material, and are a useful model for microneurosurgical training. Bovine placental vessels have some structural differences compared to human placentas, but they can be easily and continuously obtained from local dairies. Both human and bovine placentas are expansive, allowing several students to work at once on various sizes of vessels. They can be stored unperfused in normal saline in the refrigerator for up to at least a week. These models have a high face, content, and constructive validity. Models of cadaveric vessels have also been described [11]. The advantage of human cadaveric models is the possibility to practice the relevant human vessels of various sizes and diameters. However, this advantage comes with a risk of infections and need for appropriate infrastructure for acquisition and work with cadaveric material.

All of the above-mentioned models can be augmented by creating artificial perfusion using a closed system that mimics the circulation, using silicone tubes for intravenous infusion [52]. Standard infusion pumps can be connected to the system to simulate arterial pressure and pulsation may be simulated with these pumps. The system is filled with normal saline and can be tinted to achieve a resemblance to blood. A disadvantage of dyes is their frequent staining of the walls of the vessels. Blood perfusion most accurately simulates a living model due to the correspondence of viscosity and fluidity in living models, but may be impractical [53].

Perfused cadaver brain is a laborious model to set-up but is most similar to the real operative environment [54]. The opening of the arachnoid cisterns, vascular dissection, and microanastomosis can be performed very realistically in such a model although the actual “feel” of the vessel is usually altered (i.e., stiffened) as it is affected by the perfusate.

## 6. “Wet” Training Using Live Models

Microvascular training using live models could be considered as one of the best substrates for training of microvascular anastomoses due to the presence of natural blood flow. Despite the huge variety of models designed to replace training using animals, live models remain to be an indispensable part of microsurgical courses around the world.

Training conducted on laboratory animals has high indicators of face and constructive validity [8]. Working with animal vessels allows feasible rehearsal of the main stage of reconstructive vascular brain surgeries, the direct microanastomosis [55]. The presence of natural blood flow, the potential for thrombosis, and relative similarity of live animal tissues to human allow trainees to practice technique with great viability and realism.

**Figure 1:**
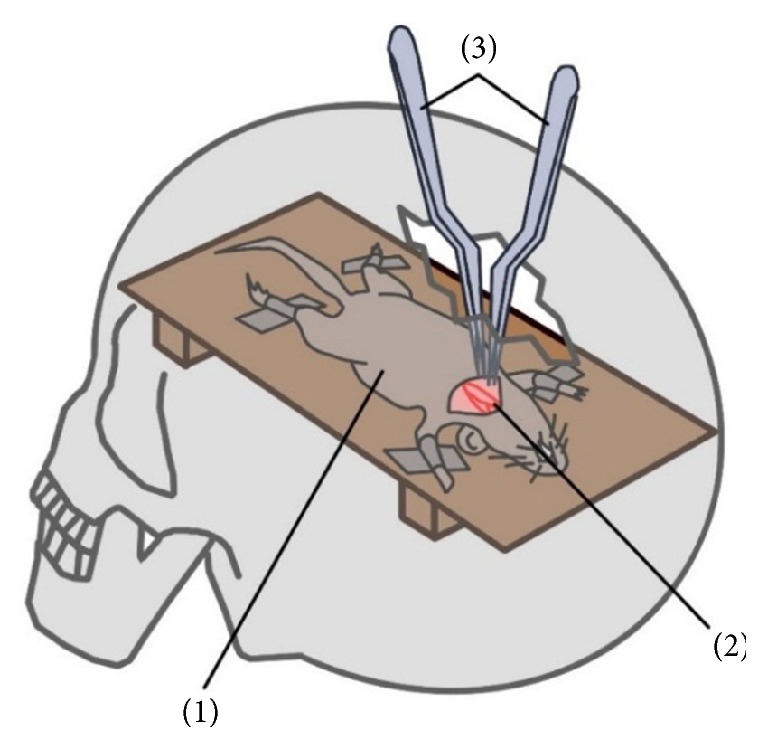
Microsurgical training on rat carotid arteries using an artificial skull with craniotomy as a simulation of surgical approach: (1) rat, (2) rat carotid arteries, and (3) microsurgical instruments.

A Wistar rat is the most commonly used laboratory animal in microvascular laboratories (Figure 1).

Microvascular suturing can be practiced on multiple large animal vessels including femoral, iliac, and carotid arteries and veins, aorta, and vena cava. Multiple types of anastomoses can be performed on the rat vessels including end-to-end, end-to-side, and side-to-side anastomoses and various arterial loops [8]. Venous [56], arterial, and synthetic vascular grafts [57] can also be used for practice on live animals.

Performance of anastomosis in the deep confined surgical field can be simulated with a plastic hand-rest that is placed above the operated area of the rat. This gives an open field about 3 cm wide and 3 cm deep. An anastomosis of the arteries and veins is then carried out through this opening [8].

Vessels of the rat are also suitable for submillimeter microanastomoses, like on the distal femoral neurovascular bundle with vessel diameter of 0.5 to 0.8 mm [58]. Vessels of such small diameter may be used for the treatment of moyamoya disease in children. However, otherwise, submillimeter vessels are rarely anastomosed in neurosurgery.

Working with living tissues provides great opportunities for training and assessing the skills of the surgeon. One of the most serious complications of the EC-IC bypass operations is cerebral ischemia associated with vascular thrombosis. The frequency of thrombosis has been reported to be as low as 1.7% in specialized centers [59]. The presence of thrombosis cannot be estimated on a nonliving model; therefore laboratory animal models give the opportunity to identify and analyze the causes of errors and develop methods to prevent their further occurrence.

Another advantage of working with animals is the ability to fully assess the damage to surrounding tissues during the operation. During training with nonliving models, the smallest damage that the surgeon does to surrounding tissues is not always noticeable. Live models can reveal such imperfections and provide valuable feedback to the operator. Also, the limitation of time is an important condition when working on anesthetized laboratory animal.

However, when working with laboratory animals, there are additional requirements for equipping the laboratory, coordinating logistics and training with procurement and handling of animals, acquiring ethical committee approval, ensuring adequate pharmaceutical control, and caring for animals during the surgery. The purchase and maintenance of laboratory animals impose serious material costs for the organization of a microvascular training laboratory. In order to minimize economic losses, various methods are being used: reducing work with animals on the initial stages and replacing them with synthetic or using tissue models from cryopreserved animal vessels left after training by more experienced trainees [60]. Trainees working with live animals should be required to practice on as many appropriate vessels as possible, not only on carotid arteries, but also on various accessible arteries and veins in the body as mentioned, even to include the vas deferens.

Despite the economic difficulties in their organization, microsurgery courses using live models have many advantages. They provide an excellent substrate to improve surgical skills and provide high accuracy of simulating clinical operations. The use of live models at advanced courses within the training program for microsurgeons is an excellent simulation model for complex procedures for microsurgical reconstruction. The live rat is one of the most universal models of microsurgery courses around the world, and 50 years after microsurgery was first developed, the laboratory rat model is still valid for developing microsurgery skills.

## 7. New Horizons in the Evolution of Microanastomosis Simulation Models

Training in microsurgery has always been conducted in the modeled environment, and the use of laboratory rat as a teaching model became standard, despite being without objective evidence of advantage over other models. The ex vivo simulation environment is becoming increasingly appealing on the basis of research that indicates reduced economic costs and limiting the use of animals. The experimental ex vivo training offers an economically efficient means to master microsurgical skills and it is in this environment that learning curves for specific exercises can be established [61]. Modern microsurgical training is often based on theories of acquiring and developing skills. As a result, validation and modeling have become increasingly important components of the microsurgical simulation.

As we proceed towards standardization of the microsurgical simulation models, there is a need to choose reliable assessment tools that adequately assess microsurgical competence (Table 2).

Requirements for objective methods for evaluating microsurgical techniques led to the development of several global rating scales. These scales split the process of microsurgery into the relevant components, which are found to contribute to the overall effectiveness of the task accomplishment [62]. Video recording allows one to assess skills in a controlled environment [63], and, as technology developed, various methods for assessing competence became available, such as hand motion analysis. For example, the Imperial College of Surgeons capture device uses motion sensors attached to hands to assess hand movements and the length of time to complete a task [64]. It was shown that this device has applications for the evaluation of trainees microsurgeons [65].

One of the future directions in the development of microsurgical techniques is the use of robotic systems. Performing operations using these devices has several advantages. For example, the presence of hand tremor during surgical procedures is completely eliminated with robots, which is important when working under high magnification [66]. Furthermore, the precision of manipulation can be increased to the level that surpass free-hand human abilities [67]. These advantages provide great promise for using robotics in microsurgical manipulations. However, the main current limitation of robotic systems is an increase in the time spent for manipulations compared to standard techniques by 24–60% [67, 68]. Robotic systems attached to the arm or hand may limit the normal movement of the hand, thus significantly slowing the procedure. When using the Zeus® robotic surgery system, the increase in the time to complete anastomosis is also associated with the large size of the needle holder installed on this device [68].

The use of robotic systems to teach microsurgical skills may play a role in the education of the future generations of microsurgeons. The advantage includes familiarization with the methods of remote robotic manipulation of the instruments at the early stage of training. With the use of a robotic system, the goal is to bridge the gap in the quality and speed of anastomoses between the experienced surgeon and a specialist who does not possess special skills in microsurgery [67]. However, the fact that anastomosis completion time is still shorter with conventional method when compared to a robotic one [68] indicates the need of substantial improvements in robotic microsurgery before its use in surgical practice or training could be approved. Incorporation of various microvascular anastomotic devices such as Precise (3M, St. Paul, MN) for end-to-end anastomosis and Elana (Elana bv, Utrecht, Netherlands) for end-to-side anastomosis would result in decrease of the anastomosis time and could advance the field.

During microsurgical procedures, the operator is visually immersed in the operative environment with a microscope thorough the eyepieces; therefore this type of surgery is well suited for developing virtual reality simulation technologies [69]. Stanford University has developed a system that consists of a graphical workstation connected to a stereoscopic display. Real microsurgical instruments, to which motion sensors are connected, are used as input devices. Traceable tools allow the user to perform microanastomoses, and the system evaluates the effectiveness of the surgeon based on a set of objective variables [70]. Further research is necessary in this area to assess the role of virtual reality in surgical education, especially for the manual skill development. However, with the rapid advances in the three-dimensional visualization and tactile feedback sensors, there is a high likelihood that such simulators would play an increasing role in surgical learning of microanastomosis techniques.

## 8. Conclusions

In this article, we reviewed several models for the study and training of vascular microsurgery. Although very few of these models have been validated, such become an increasingly important component of surgical education. Additionally, evaluation of microsurgical skills is becoming an important part of the current evaluation of the trainee's competence in a training program. At more advanced levels, simulation models can be utilized to assess the level of competence of surgeons within the recertification process. It is important that such evaluation be based on validated models.

Modern technologies provide highly accurate and extremely realistic learning environments. Microsurgery, over the past decades, has made significant progress in the field of materials, technologies, and the scope of reconstructive methods. However, efforts are yet to be made to establish appropriate tools and models for training and assessment of competence. Evidence of reliability and validity must be obtained before models can be included in training and accreditation programs. The ideal model should be one that can combine being highly relevant and meaningful, having high constructive and predictive validity, and being economically efficient. Implementation of validated realistic training models and modern objective assessment tools will further advance the microsurgical training.

## Figures and Tables

**Table 1:** Validated models for microvascular anastomosis practice.

Type	Author	Model	Approximate cost, availability, logistics	Validity
Non-animal	Meier et al., 2004 [5]	Silicone and polymer microtubes	$10–100 cost, no need for coordination with the ethics committee, simple technical equipment	High constructive validity, low face validity
	Guler and Rao, 1990 [6]	Surgical glove	No cost, no need for coordination with the ethics committee, simple technical equipment	High constructive validity, low face validity

Animal	Bates et al., 2013 [7]	Poultry blood vessels	Little to moderate cost, no need for coordination with the ethics committee, simple technical equipment	High constructive validity, low face validity
	Shurey et al., 2014 [8]	Live laboratory animals (rat)	Average cost $120, requires protocol approval by the Institutional Animal Care and Use Committee, requires vivarium and animal care staff, requires pharmaceuticals and training for administration of anesthesia	High face and constructive validity
	Belykh et al., 2016 [9]	Bovine placental arteries	No to little cost, anatomical relevance, requires coordination with local dairy. Does not require approval from ethics committee	High construct, content, and constructive validity

Human	Aboud et al., 2002 [10]	Perfused cadavers	High cost of cadaveric material, requires special laboratory space to work with cadaver material, normal vessel structure and feel usually disrupted by preservation techniques	No formal validation performed
	Tellioglu et al., 2009 [11]	Human cadaveric vessels	High cost of cadaveric material, preservation techniques, time or temperature changes may damage vessel structure and feel, requires special laboratory space to work with cadaver material	No formal validation performed
	Belykh et al., 2016 [9]	Human placental arteries	No cost, anatomical relevance, requires coordination with obstetrics department, requires protocol approval from the ethics committee	High construct, content, and constructive validity

Technical	Alrasheed et al., 2014 [12]	Robotic systems	High cost of equipment and services	No formal validity of microscope or robot

**Table 2:** Tools for microvascular anastomosis performance assessment.

Author	Assessment Tool	Comments
Martin et al., 1997 [13]	Objective structured assessment of technical skills	Established validity for general surgery and microsurgery, this is one of the first and most reliable assessment tools

Grober et al., 2003 [14]	Imperial College surgical assessment device	Motion tracking device attached to the surgeon's hands for objective assessment of the motions during surgical training

Moulton et al., 2006 [15]	Modified objective structured assessment of technical skills	A video based modified version of the objective structured assessment of technical skills

Chan et al., 2010 [16]	Structured assessment of microsurgery skills	Validated tool for microanastomosis assessment validated in clinical setting

Temple and Ross, 2011 [17]	University of Western Ontario microsurgical skills acquisition/assessment instrument	Validated tool for microvascular performance assessment

Ghanem et al., 2013 [18]	Anastomotic patency assessment tool	This tool uses qualitative photogrammetry for the assessment of anastomosis quality with a special attention to the intimal surface

Alrasheed et al., 2014 [12]	Structured assessment of robotic microsurgical skills	Tool was developed to assess microsurgical skills using the Da-Vinci surgical robot while performing a 3-mm vessel diameter microanastomosis

Satterwhite et al., 2014 [19]	The Stanford microsurgery and resident training scale	This tool was validated on latex and chicken vessels

Aoun et al., 2015 [20]	Northwestern objective microanastomosis assessment tool	Face and construct validity has been established for this tool

Harada et al., 2015 [21]	Instrument motion tracking	Infrared motion tracking camera has been use for objective measurement of the instrument movements in experienced and novice surgeons during microanastomosis training

Ghanem et al., 2016 [22]	Anastomosis lapse index	A list of possible errors could be used for quantitative assessment

Belykh et al., 2017 [23]	Objective structured assessment of aneurysm clipping skills	Tool was validated on the human placenta aneurysm models
